# Transanal total mesorectal excision port-assisted perineal hernia repair: A case report

**DOI:** 10.3389/fonc.2022.1036145

**Published:** 2022-10-18

**Authors:** Xudong Peng, Yinggang Ge, Jianwen Zhang, Zhengqiang Wei, Hongyu Zhang

**Affiliations:** Gastrointestinal Surgical Unit, The First Affiliated Hospital of Chongqing Medical University, Chongqing, China

**Keywords:** perineal hernia, repair, TaTME, laparoscopy, apr

## Abstract

Perineal hernia after abdominoperineal resection (APR) is a troublesome problem, and severe cases require surgical treatment. However, perineal hernia repair is challenging, especially when combined with intestinal adhesions. The difficulty of the operation lies in performing adhesiolysis and mesh placement under poor visibility. While there are traditional, laparoscopic and even robotic methods of performing this procedure, no easy and minimally-invasive approach has been reported. Here, we report the case of a patient with perineal hernia, who underwent transanal total mesorectal excision (TaTME) port-assisted laparoscopic perineal hernia repair. The operation was successful, the postoperative recovery was uneventful, the patient’s symptoms improved significantly, and no recurrence was found during the 4-month follow-up. The availability and safety of TaTME port-assisted perineal hernia repair provide a promising approach for hernia repair. Compared with traditional perineal or laparoscopic abdominal approaches, this procedure is less invasive and results in a better field of vision.

## Background

Perineal hernia is a rare hernia, which typically occurs after abdominoperineal resection (APR) and pelvic exenteration (1). The reported incidence of perineal hernia varies widely from 0.2% to 27% (2, 3). Most patients with perineal hernia receive conservative treatment, but a few patients with obvious symptoms need surgical treatment. Pelvic floor hernia repair is challenging, especially when combined with intestinal adhesion. Typical approaches include the transperineal approach, transabdominal approach or abdominoperineal approach (4, 5). The advantage of the transabdominal approach is that the anatomical perspective conforms to the usual surgical habits and any tumor recurrence can be detected. However, when there is a stoma, the risk of mesh infection is high (6). The transperineal approach is less invasive and placement of the mesh is relatively safe because the surgical field is far away from the stoma. The disadvantage is that the visual field is poor, and if the adhesions are heavy, the operation is extremely difficult. Therefore, some patients need to undergo an abdominoperineal operation, but this method is traumatic and complicated. The recurrence and complication rate are reported to be high in both approaches, and unrelated to the choice of the repair approach (7).

One of the authors is a participant in the colorIII clinical trial and has some experience in the use of TaTME for rectal cancer (8). Combined with our own experience, our team explored a new method of perineal hernia repair, which involved using a TaTME port to release pelvic floor adhesions through a perineal approach, and then fixing mesh. In the present case, we successfully treated a perineal hernia after laparoscopic APR by employing a TaTME port-assisted perineal approach.

## Case presentation

**Figure 1:**
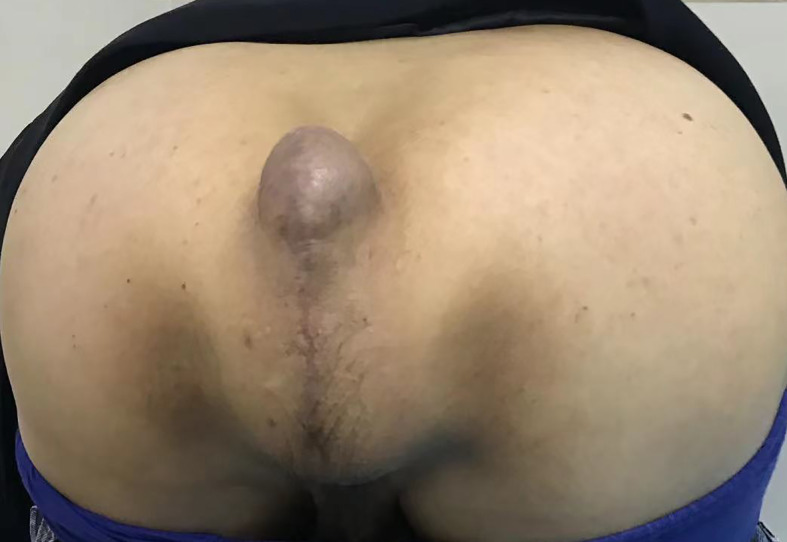
Image of the hernia preoperatively.

**Figure 2:**
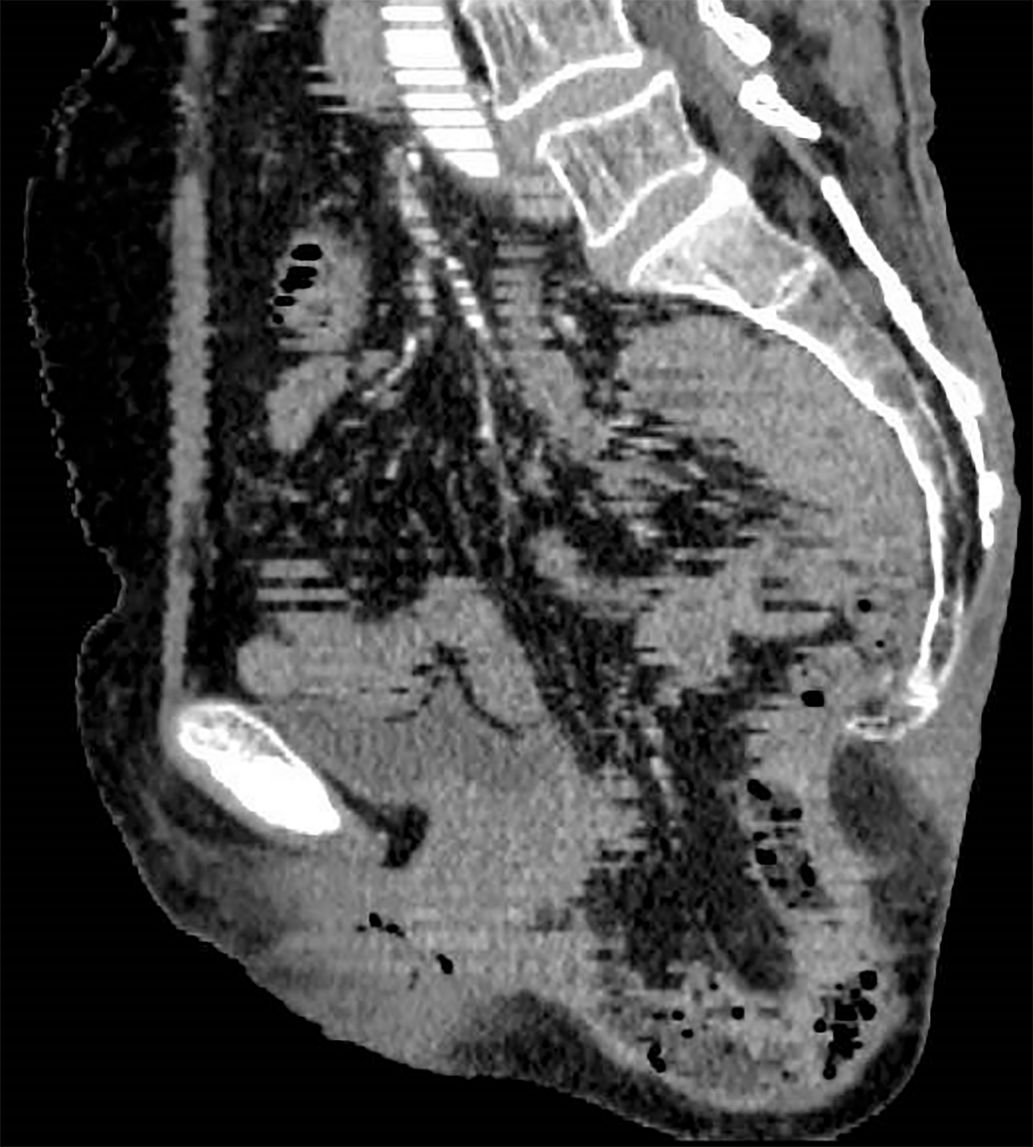
CT view of the hernia.

A 68-year-old woman with a history of hysterectomy underwent an APR operation for rectal cancer in 2021, and the pathological stage was T2N1M0. Four months later, she returned to our hospital with a complaint of bearing-down pain and bulging in the perineal region. In the knee–chest position, the size of the entire bulge was about 10 × 12 cm, which increased when holding breath and increasing abdominal pressure, whereupon the entire bulge became larger and a more obvious bulge of about 4 × 5 cm appeared in the upper part. The bulge could be partly reduced manually but not fully recovered, which suggested perineal hernia accompanied by intestinal adhesions (**Figure 1**). A contrast-enhanced CT scan confirmed the presence of a perineal hernia and ruled out cancer recurrence (**Figure 2**). Because the patient presented with obvious pain and a large bulge, surgery was necessary. However, the patient was diagnosed with thrombocytopenia, with a minimum platelet count of 30 × 10^9^/L, so she was discharged and treated with oral drugs to raise the platelet count. Seven months later, the patient’s platelet count had increased to 80 × 10^9^/L. She was readmitted to hospital and underwent TaTME platform-assisted perineal hernia repair.

After successful general anesthesia, the patient was placed in the Trendelenburg position. The laparoscopy display screen was placed next to the patient’s left shoulder. The chief surgeon was seated between the patient’s legs and the assistant holding the laparoscope stood on the right side of the chief surgeon. A longitudinal incision of approximately 6 cm was made along the previous surgical scar, and the descending pelvic floor peritoneum could be seen after the skin and subcutaneous tissue were cut. After careful incision of the peritoneum and release of the adhesions below the incision under direct vision, a large gauze was placed into the pelvic cavity to block the small intestine.

**Figure 3:**
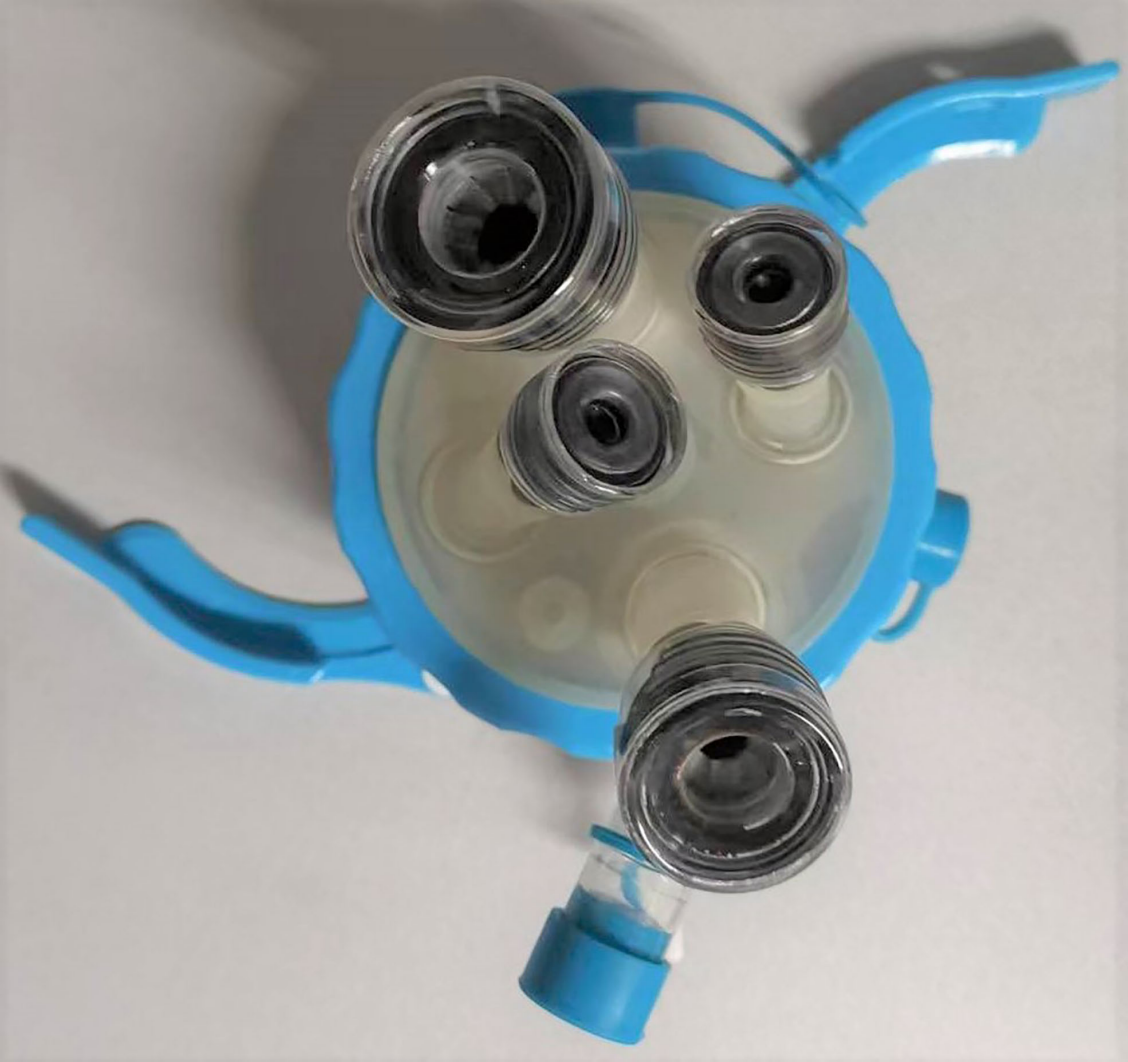
The TaTME port, which was equipped with four operating trocars and one observation trocar.

**Figure 4:**
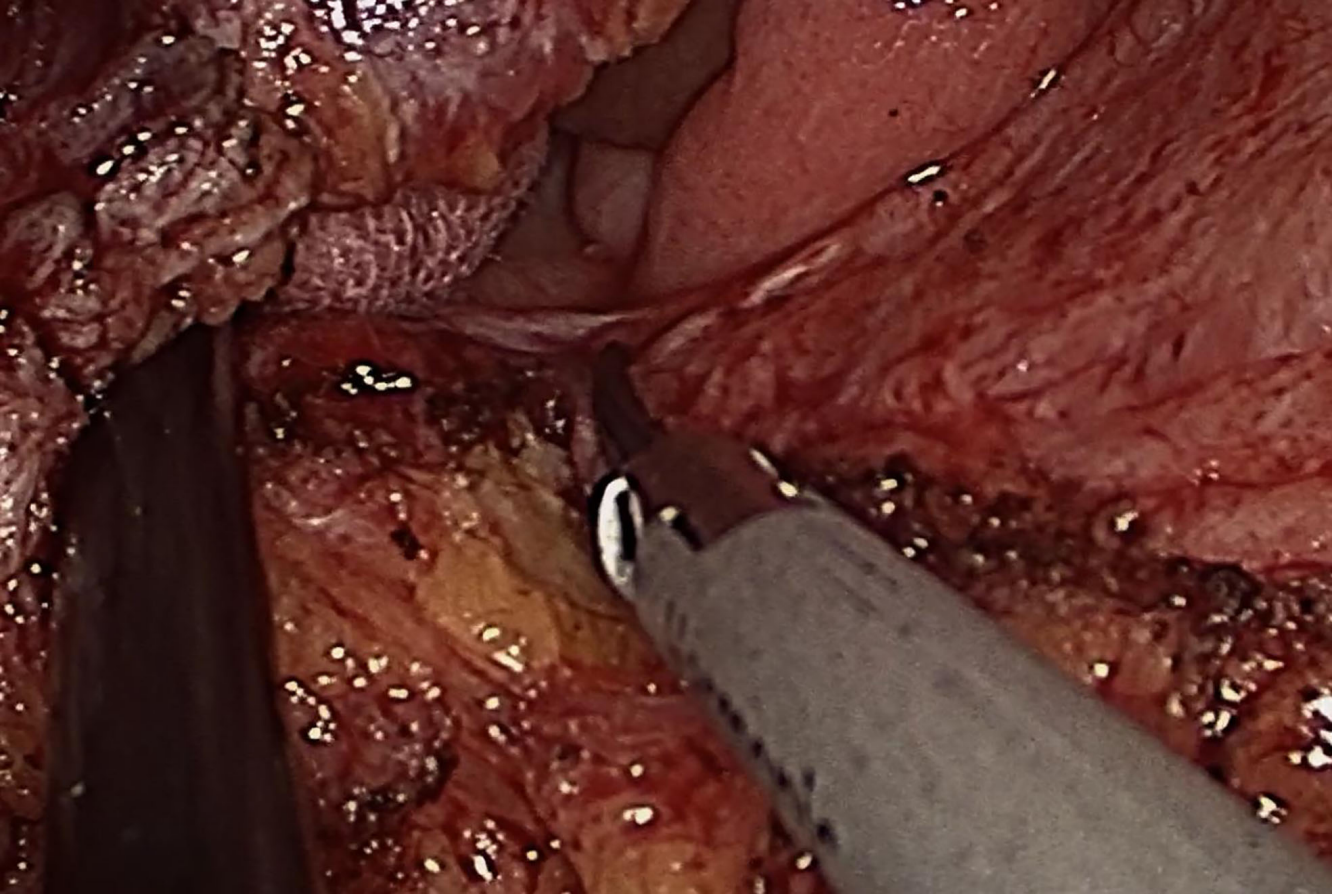
Adhesions were released using an ultrasonic knife and an electrocoagulation hook.

Next, the TaTME port, which was equipped with four operating apertures and one observation aperture, was fixed *via* the wound (**Figure 3**). The carbon dioxide pneumoperitoneum pressure was set at 12 mmHg. A 30 oblique-viewing rigid endoscope was inserted into a 10 mm trocar. The remaining 12 mm trocar and two 5 mm trocars were used as operating apertures. When the pelvic cavity was observed under the laparoscope, multiple adhesions were found between the small intestine, the mesentery and the pelvic peritoneum. Under laparoscopic view, the small intestine was pushed to the cephalic side as much as possible and the adhesions were released using a harmonic^®^ scalpel (Ethicon Inc., Cincinnati, OH, USA) and an electrocoagulation hook (**Figure 4**).

**Figure 5:**
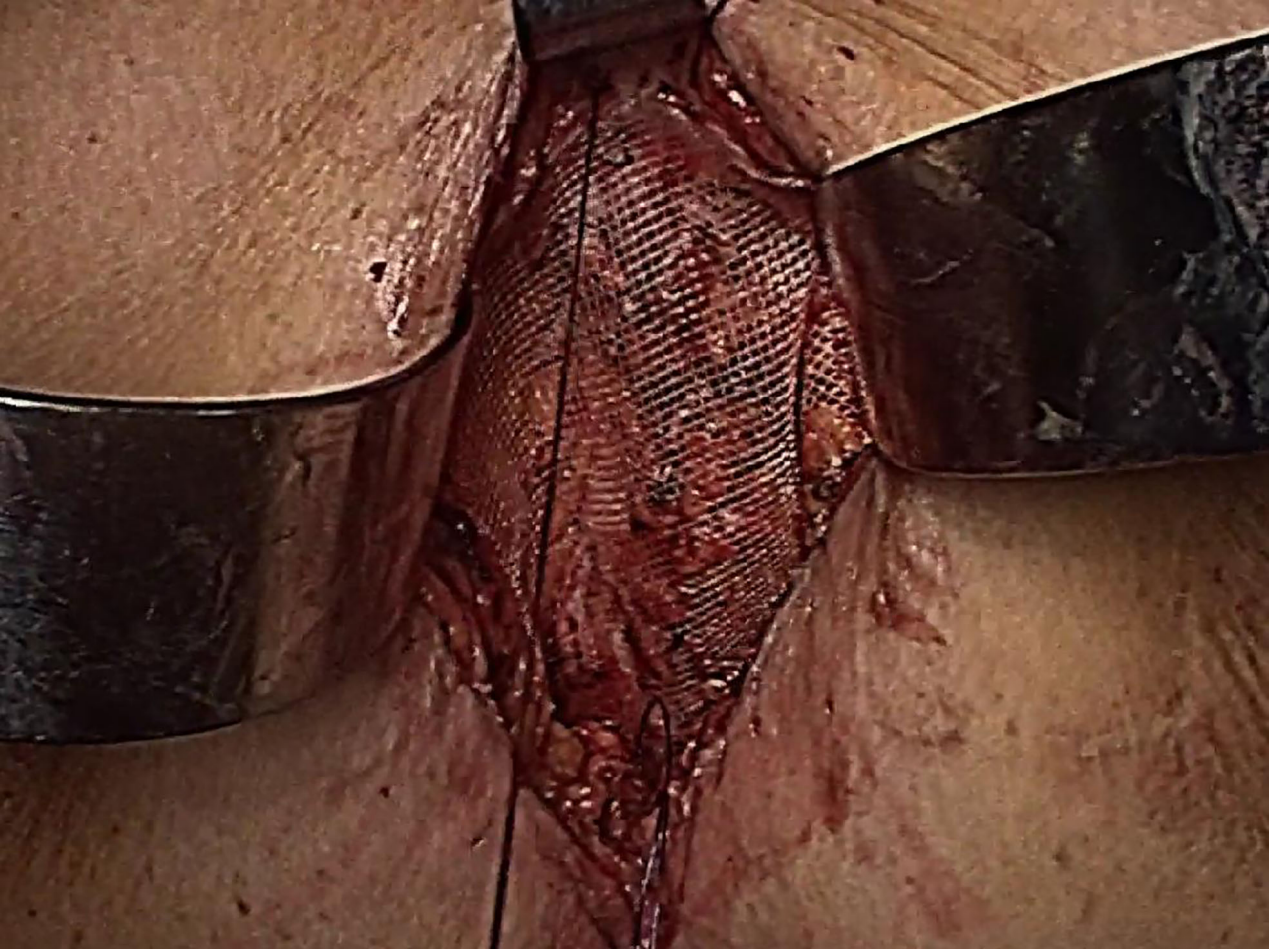
Placement of the mesh.

**Figure 6:**
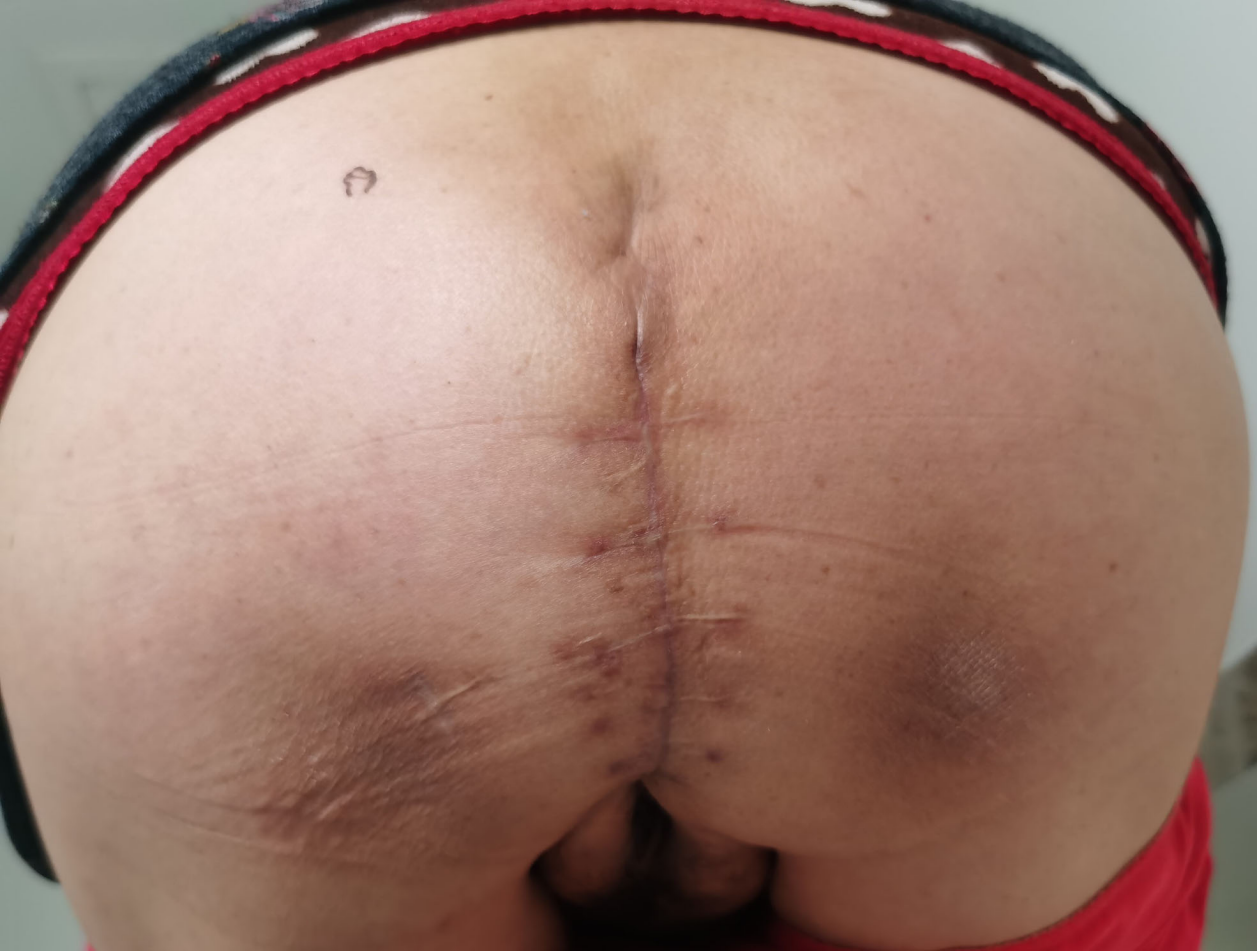
Image of the hernia postoperatively.

**Figure 7:**
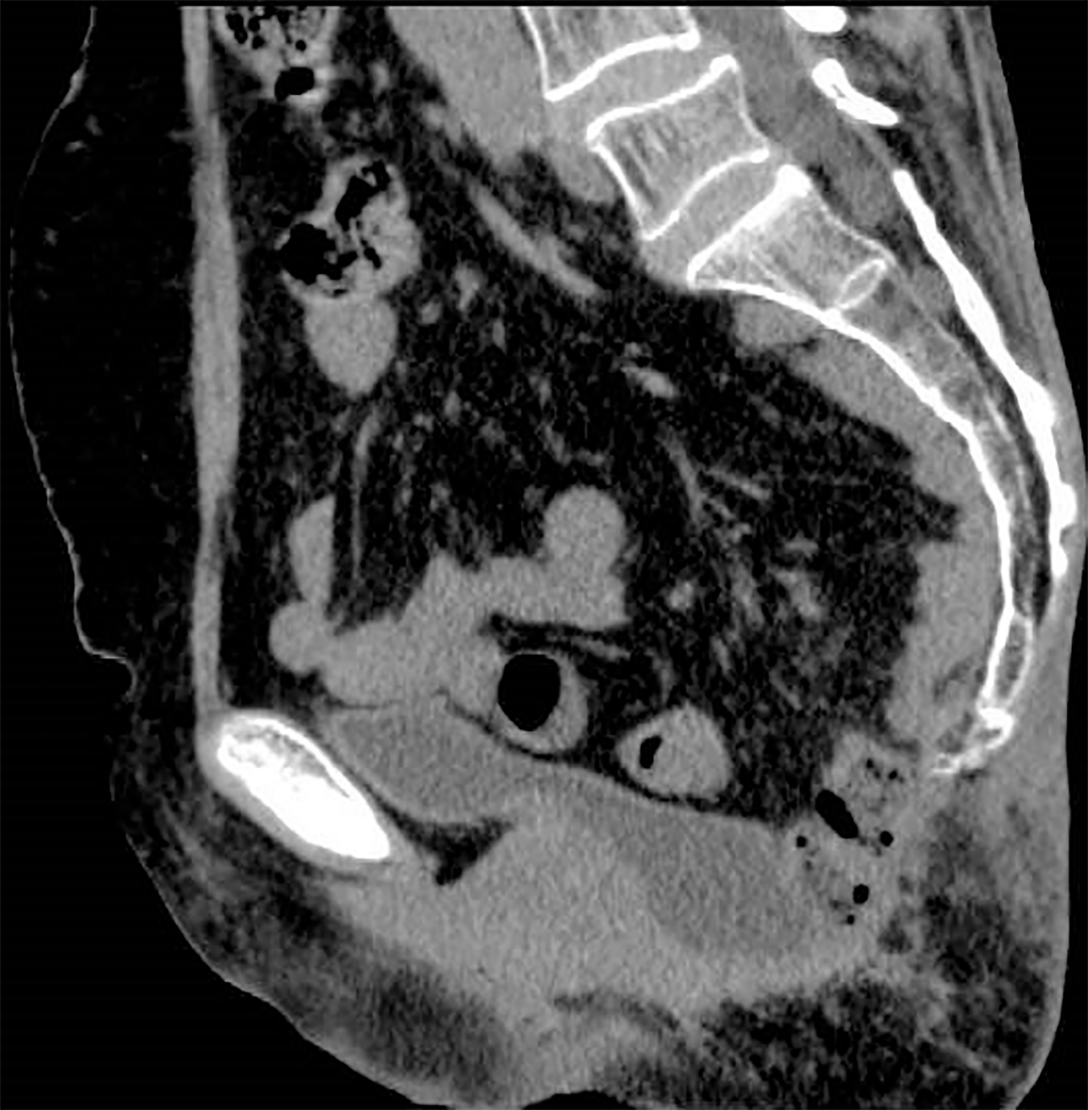
CT view of the hernia postoperatively.

**Figure 8:**
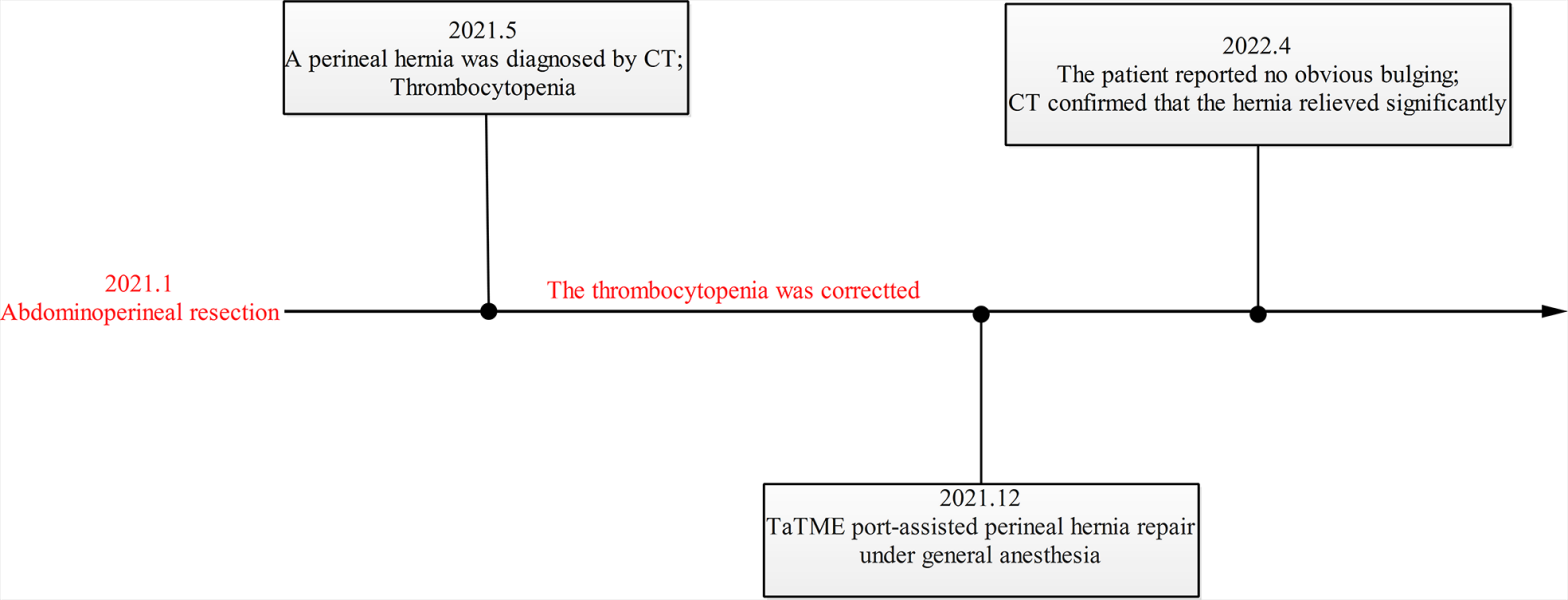
The timeline of this case.

Next, the TaTME port was removed, part of the pelvic floor peritoneum was trimmed, and the hernia sac was sutured intermittently with 2-0 absorbable sutures with a little tension. A thin polypropylene mesh (TiLENE Mesh 6000677, pfm medical AG, Cologne, Germany) was placed on the surface of the pelvic floor peritoneum and was fixed with a continuous suture using Prolene suture under direct vision. The back of the mesh was sutured to the anococcygeal ligament, the front was sutured to the posterior wall of the vagina, and both sides were sutured to the levator (**Figure 5**). The subcutaneous fat and skin were then sutured in turn. No complications occurred during or after the operation, and the patient was discharged on the seventh day after surgery. At the first follow-up examination 4 months postoperatively, the patient reported no obvious bulging and had experienced no symptoms (**Figure 6**). Meanwhile, CT revealed that although there were still bowels falling into the pelvis, the extent was significantly less than before (**Figure 7**). The timeline of this case is showed in **Figure 8**.

## Discussion

APR is an important surgical procedure for radical resection of rectal cancer, which is suitable for patients with a tumor close to the anus with a late local stage. APR involves resection of the internal and external sphincter, mesorectum and surrounding tissue, and severing of the levator ani muscle. Due to the lack of pelvic floor tissue and inaccurate perineal suturing under limited vision, perineal hernias occur in some patients. Other than surgery, risk factors for perineal hernia include neoadjuvant radiotherapy, age, and female sex (9–11). Typical clinical manifestations of a perineal hernia may include a sensation of fullness in the perineum area, and perineal bulging and pain that may only become noticeable with the Valsalva maneuver. As with other abdominal wall hernias, complications may include intestinal obstruction and bowel strangulation and perforation. However, given that patients are typically minimally- or asymptomatic, and the significant risks associated with perineal hernia repair such as mesh erosion, fistulization and chronic infection, as well as a high recurrence rate, the majority of cases are managed conservatively.

The approaches for perineal hernia repair include transperineal, abdominal and combined approaches, and surgical techniques include traditional open surgery and laparoscopic surgery (4, 12). Regardless of the approach and technique used, the procedures are primary sutures, mesh placement, and muscle flap reconstruction (13). Compared with open surgery, laparoscopy can provide an adequate field of vision. More and more publications have reported the application of laparoscopy in perineal hernia repair. Thus the evidence supports a laparoscopic approach for perineal hernia repair, but it is difficult to release adhesions and suture mesh under laparoscopic view. Li et al. reported that robot-assisted laparoscopic surgery provides a good surgical visual field and precise operation, and improves the ease of suturing, mesh positioning, and access to hard-to-reach areas (14). However, considering the economic level of developing countries, robot-assisted laparoscopic surgery is unlikely to be widely adopted.

In this case report we describe our use of TaTME platform-assisted laparoscopy for repair of a perineal hernia. This approach provided significantly improved visualization of the hernia and pelvic space, which facilitated transperineal adhesiolysis, as well as placement of mesh with wide overlap and suture fixation under direct visualization. In addition, we speculate that when the operation is difficult due to complicated adhesions, a combination of transperineal and transabdominal laparoscopy may also be used. Of course, this surgical approach also has some limitations. For example, similar to single-port laparoscopy, it is difficult for the surgeon to operate due to the lack of traction and exposure provided by an assistant. Although this patient had a smooth recovery, the short follow-up time meant that any longer-term issues have not yet come to light. In future, a randomized controlled trial will be necessary to prove whether the recurrence rate and complication rate differ between this approach and conventional repair methods.

## Conclusion

To repair this case of perineal hernia, we released pelvic floor adhesions by TaTME port-assisted laparoscopy, and then sutured the mesh under direct vision. The operation was novel and smooth without any complications. The follow-up confirmed that the short-term effect was acceptable, and the long-term effect remains to be further observed. TaTME port-assisted perineal hernia repair can benefit from the respective advantages of laparoscopic technology and the transperineal approach and the learning curve is relatively short.

## Data availability statement

The original contributions presented in the study are included in the article/**Supplementary Material**. Further inquiries can be directed to the corresponding author.

## Ethics statement

The studies involving human participants were reviewed and approved by The ethics committee of the First Affiliated Hospital of Chongqing Medical University. The patients/participants provided their written informed consent to participate in this study. Written informed consent was obtained from the individual(s) for the publication of any potentially identifiable images or data included in this article.

## Author contributions

XP, YG, JZ and HZ have made substantial, direct, and intellectual contributions to the work and agree to its publication. All authors contributed to the article and approved the submitted version.

## Funding

This study was supported by Chongqing key diseases Research and Application Demonstration Program from Chongqing Municipal Health Commission (Colorectal Cancer Prevention and Treatment Technology Research and Application Demonstration [No. 2019ZX003].

## Acknowledgments

We thank International Science Editing (http://www.internationalscienceediting.com) for editing this manuscript.

## Conflict of interest

The authors declare that the research was conducted in the absence of any commercial or financial relationships that could be construed as a potential conflict of interest.

## Publisher’s note

All claims expressed in this article are solely those of the authors and do not necessarily represent those of their affiliated organizations, or those of the publisher, the editors and the reviewers. Any product that may be evaluated in this article, or claim that may be made by its manufacturer, is not guaranteed or endorsed by the publisher.
